# Xp11.22 deletions encompassing *CENPVL1*, *CENPVL2*, *MAGED1* and *GSPT2* as a cause of syndromic X-linked intellectual disability

**DOI:** 10.1371/journal.pone.0175962

**Published:** 2017-04-17

**Authors:** Christina Grau, Molly Starkovich, Mahshid S. Azamian, Fan Xia, Sau Wai Cheung, Patricia Evans, Alex Henderson, Seema R. Lalani, Daryl A. Scott

**Affiliations:** 1 Department of Molecular and Human Genetics, Baylor College of Medicine, Houston, Texas, United States of America; 2 Baylor Genetics, Houston, Texas, Unite States of America; 3 Departments of Pediatrics and Neurology, University of Texas Southwestern Medical School, Dallas, Texas, United States of America; 4 The Newcastle upon Tyne Hospitals, Newcastle upon Tyne, England; 5 Department of Molecular Physiology and Biophysics, Baylor College of Medicine, Houston, Texas, United States of America; TNO, NETHERLANDS

## Abstract

By searching a clinical database of over 60,000 individuals referred for array-based CNV analyses and online resources, we identified four males from three families with intellectual disability, developmental delay, hypotonia, joint hypermobility and relative macrocephaly who carried small, overlapping deletions of Xp11.22. The maximum region of overlap between their deletions spanned ~430 kb and included two pseudogenes, *CENPVL1* and *CENPVL2*, whose functions are not known, and two protein coding genes—the G1 to S phase transition 2 gene (*GSPT2*) and the MAGE family member D1 gene (*MAGED1*). Deletions of this ~430 kb region have not been previously implicated in human disease. Duplications of *GSPT2* have been documented in individuals with intellectual disability, but the phenotypic consequences of a loss of *GSPT2* function have not been elucidated in humans or mouse models. Changes in *MAGED1* have not been associated with intellectual disability in humans, but loss of MAGED1 function is associated with neurocognitive and neurobehavioral phenotypes in mice. In all cases, the Xp11.22 deletion was inherited from an unaffected mother. Studies performed on DNA from one of these mothers did not show evidence of skewed X-inactivation. These results suggest that deletions of an ~430 kb region on chromosome Xp11.22 that encompass *CENPVL1*, *CENPVL2*, *GSPT2* and *MAGED1* cause a distinct X-linked syndrome characterized by intellectual disability, developmental delay, hypotonia, joint hypermobility and relative macrocephaly. Loss of GSPT2 and/or MAGED1 function may contribute to the intellectual disability and developmental delay seen in males with these deletions.

## Introduction

Xp11.22 comprises approximately 5 Mb of DNA (chrX:49,800,001–54,800,000, hg19). A number of pathogenic deletions and duplications involving Xp11.22 have been described in individuals with developmental delay, intellectual disability and/or autism [1–14]. These phenotypes have been attributed to changes in the copy number of several genes including, *HUWE1*, *KDM5C*, *IQSEC2*, *TSPYL2*, *SHROOM4*, *PHF8* and *FAM120C*.

Mutations in *HUWE1* are the cause of mental retardation, X-linked syndromic, Turner type [Online Mendelian Inheritance in Man (OMIM, http://www.ncbi.nlm.nih.gov/omim) #300706], Juberg-Marsidi syndrome [OMIM #309580] and Brooks-Wisniewski-Brown syndrome [OMIM #300612] [5,15–17]. Xp11.22 microduplication syndrome [OMIM #300705] is caused by duplication of *HUWE1* and is characterized by mild to moderate intellectual disability [5].

Duplications and deletions involving *KDM5C*, *IQSEC2* and *TSPYL2* that do not overlap with *HUWE1*, have also been shown to cause developmental delay, intellectual disability, behavioral disturbances and/or autistic features [11,12]. In addition, mutations of *KDM5C* cause mental retardation, X-linked, syndromic, Claes-Jensen type [OMIM #300534] and mutations of *IQSEC2* cause mental retardation, X-linked 1 [OMIM #309530] [18,19]. Although mutations of *TSPYL2* have not been associated with a particular human phenotype, TSPYL2 interacts with CASK whose gene is mutated in mental retardation, with or without nystagmus, FG syndrome 4 [OMIM #300422] and mental retardation and microcephaly with pontine and cerebellar hypoplasia [OMIM #300749] [20–23].

Armeanet et al. identified a male with developmental delay and intellectual disability who carried an Xp11.22 deletion involving *SHROOM4* and intellectual disability and autism has been described by Qiao et al. and De Wolf et al. in males carrying Xp11.22 deletions that included *PHF8* and *FAM120C* [1,6,9]. Mutations of *SHROOM4* have been reported to cause Stocco dos Santos X-linked mental retardation syndrome [OMIM #300579] and mutations of *PHF8* cause mental retardation syndrome, X-linked, Siderius type [OMIM # 300560] [24–28]. Although mutations of *FAM120C* have not been associated with a particular human phenotype, this gene has been proposed as a positional candidate gene for autism based on its expression pattern in the brain and FAM120C’s interaction with CYFIP1, an important binding partner of the fragile X mental retardation protein (FMRP) [6,29].

We describe four males (subjects 1–4) from three families who carry maternally inherited, partially overlapping Xp11.22 deletions that encompass two pseudogenes, *CENPVL1*, *CENPVL2*, whose functions are not known, and two protein coding genes—the G1 to S phase transition 2 gene (*GSPT2*) and the MAGE family member D1 gene (*MAGED1*). These deletions do not include *HUWE1*, *KDM5C*, *IQSEC2*, *TSPYL2*, *SHROOM4*, *PHF8* or *FAM120C*. Subjects 1–4 have a common constellation of findings which includes developmental delay, intellectual disability, hypotonia, joint hypermobility and relative macrocephaly. Short stature and failure to thrive were also common.

## Materials and methods

### Subject identification and accrual

Subjects 1 and 2 were identified by searching a clinical database of individuals referred to Baylor Genetics for copy number variant (CNV) analysis. Subjects 3 and 4 are siblings who were identified through a search of the DECIPHER database (https://decipher.sanger.ac.uk/; patient 276715). All families provided written informed consent for their inclusion in this study. This consent was obtained using consent forms approved by the Institutional Review Board of Baylor College of Medicine.

The family of subject 1 has given written informed consent (as outlined in PLOS consent form) to publish his case details and photographs.

### Array-based CNV analyses

Array-based CNV analyses were performed for subjects 1 and 2 on a clinical basis at Baylor Genetics. These analyses included a custom-designed array comparative genomic hybridization (aCGH) analysis using 400,000 probes, for the detection of gains or losses of genomic material, and a single nucleotide polymorphism (SNP) analysis using 60,000 probes, for the detection of uniparental disomy (UPD) and absence of heterozygosity (AOH).

### X-inactivation studies

A HUMARA assay was used to determine the X-inactivation pattern in the mother of subject 1 [30]. Briefly, extracted DNA was treated with the methylation-sensitive restriction enzyme HpaII followed by PCR amplification of the CAG repeats in the *AR* gene. In this assay, random X chromosome inactivation is demonstrated by the generation of two PCR bands of similar intensity.

## Results

**Fig 1:**
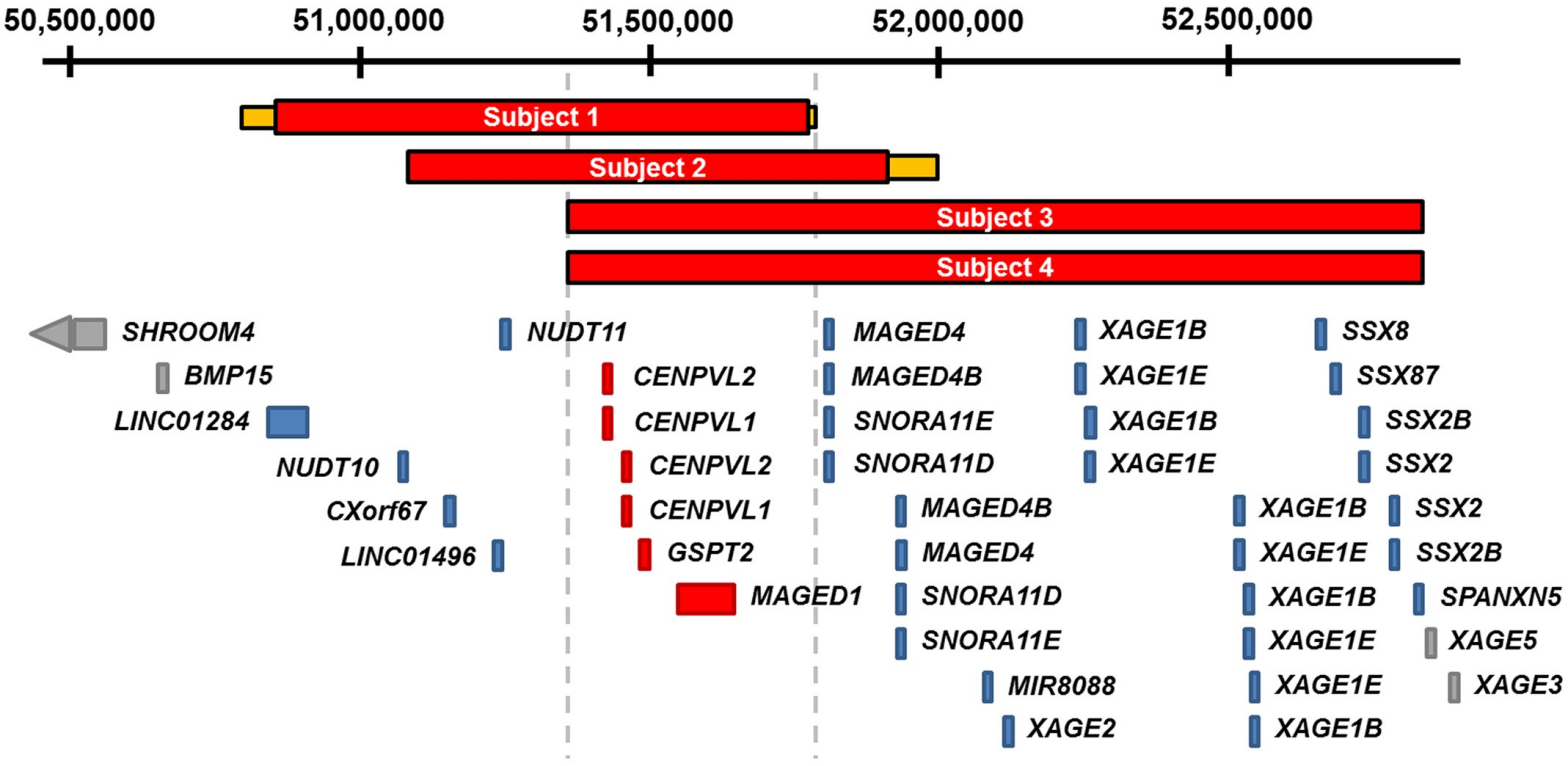
Schematic representation of the Xp11.22 deletions carried by subjects 1–4. The minimum (red) and maximum (yellow) deletions of each subject are shown in relation to the positon of Xp11.22 genes. The coordinates shown at the top of the figure are based on hg19. The maximum region of overlap is represented as dashed gray lines. The RefSeq genes located in this critical region—*CENPVL1*, *CENPVL2*, *GSPT2* and *MAGED1*—are shown in red. Genes depicted in blue are deleted in a subset of subjects 1–4. Genes depicted in gray were not deleted in subjects 1–4.

By searching a clinical database of >60,000 individuals referred for CNV analyses, we identified two males (subjects 1–2) that were hemizygous for relatively small (< 2 Mb) Xp11.22 deletions involving *CENPVL1*, *CENPVL2*, *MAGED1* and *GSPT2*. A search of the DECIPHER database (https://decipher.sanger.ac.uk/), revealed two brothers (subjects 3 and 4) who carried partially overlapping Xp11.22 deletions that also involved these genes. All of these individuals inherited their deletions from their asymptomatic mothers. The molecular findings and clinical phenotypes of these subjects are summarized below and in Fig 1 and Table 1.

**Table 1:** Molecular findings and clinical phenotypes of subjects 1–4.

	Subject 1	Subject 2	Subject 3	Subject 4
Xp11.22 Deletion Minimum (hg19)	chrX:50,847,688–51,773,705	chrX:51,079,343–51,912,188	chrX:51,357,052–52,838,176	chrX:51,357,052–52,838,176
Xp11.22 Deletion Maximum (hg19)	chrX:50,789,912–51,786,912	chrX:51,079,341–51,990,483	ND	ND
Age, gender	3-year-8-month-old male	7-year-9-month-old male	6-year–old male	4-year-old male
Intellectual disability/developmental delay	+	+	+	+
Hypotonia	+	+	+	ND
Joint hypermobility	+	+	+	+
Relative macrocephaly	+	+	+	+
Other growth problems	None	Failure to thrive	Failure to thrive, short stature	Short stature
Other medical problems	Congenital muscular torticollis, laryngomalacia, right-sided cryptorchidism, right-sided inguinal hernia, gastroesophageal reflux disease, food allergies	Gastroesophageal reflux disease	Hypermetropia, intermittent exotropia, arthralgias	Exotropia, amblyopia
Other physical exam findings	Small testes, hypoplastic scrotum, medial malleolar displacement, sandal gap	Pes planus, medial malleolar displacement		Epicanthal folds, capillary nevus, pes planus

ND = Not documented

**Fig 2:**
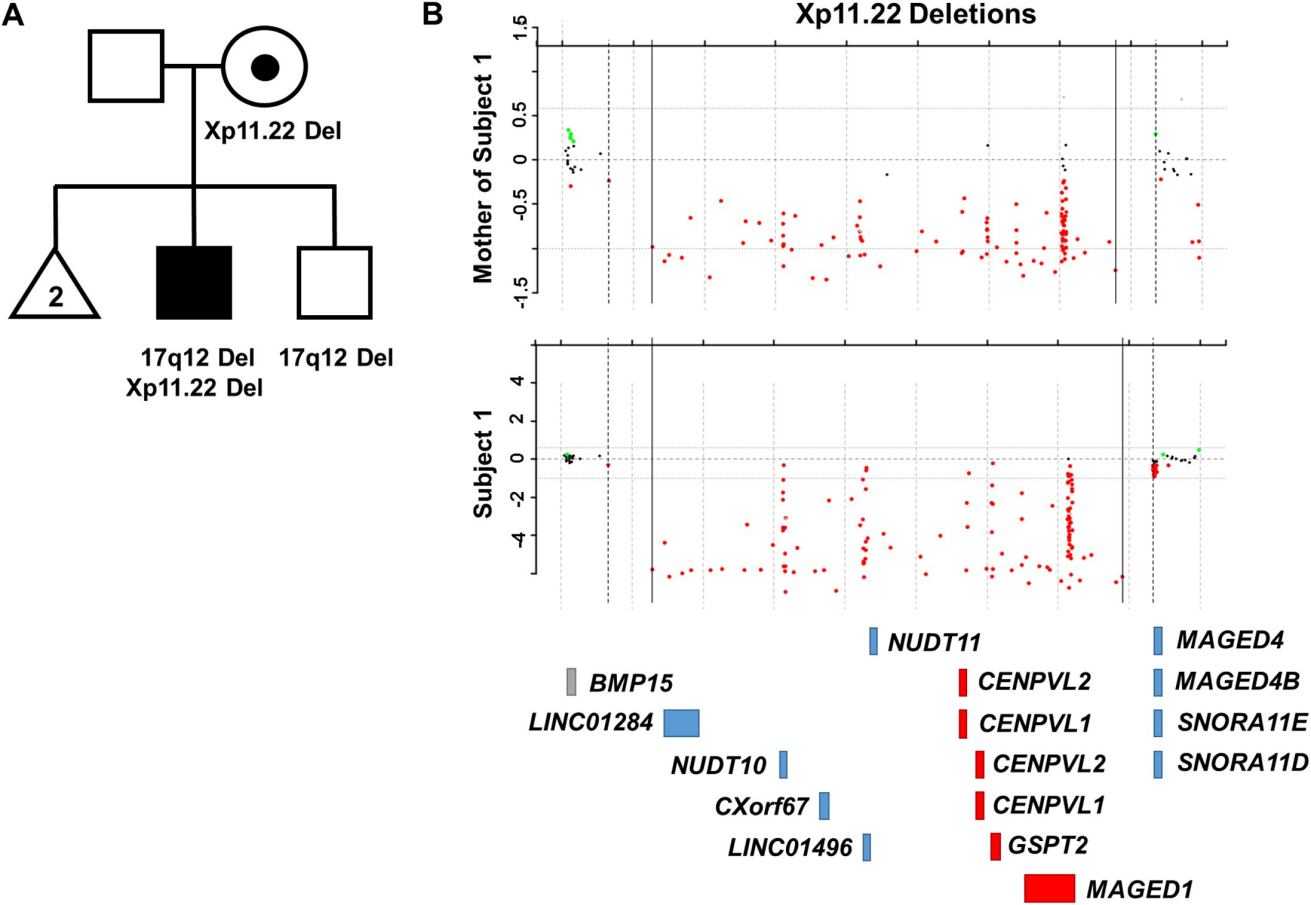
Pedigree and array-based CNV analysis for subject 1. (A) Subject 1 inherited an interstitial Xp11.22 deletion from his asymptomatic mother. His unaffected brother does not carry this deletion. Both subject 1 and his unaffected brother carry a maternally-inherited 17q12 deletion. (B) Data from the array-based CNV analyses performed on the subject 1 and his mother. The approximate locations of the RefSeq genes in this region are shown below. Genes depicted in red are those found in the maximal overlapping region encompassed by the deletions of subjects 1–4. Genes depicted in blue were deleted in a subset of subjects 1–4. Genes depicted in gray were not deleted in subjects 1–4.

Subject 1 is a 3-year-8-month-old male of Northern European descent who carries an ~1 Mb Xp11.22 deletion (minimum deletion chrX:50,847,688–51,773,705; maximum deletion chrX:50,789,912–51,786,912; hg19) that he inherited from his asymptomatic mother (Fig 2). This deletion was confirmed using a series of PCR primers designed to amplify unique regions of Xp11.22 (S1 Table). Studies performed on the maternal sample did not show evidence of skewed X-inactivation. His asymptomatic brother was not found to carry this deletion.

Subject 1 also carried a small, maternally-inherited deletion of a non-disease-associated region on chromosome 17q12 (minimum deletion chr17:33,682,606–33,760,379; maximum deletion chr17:33,665,134–33,772,686, hg19). This deletion involves two genes, *SLFN11* and *SLFN12* (OMIM #614953, #614955). These genes encode members of the SLFN family of proteins that function to modulate T-cell development and activation [31,32]. This 17q12 deletion is also carried by subject 1’s asymptomatic brother.

Subject 1 was conceived naturally by non-consanguineous parents. His family history was notable for two previous miscarriages. He was born at 39 weeks gestation via cesarean section due to a failure to progress. His birth weight was 3.675 kg (75^th^ centile) and his length as was 54 cm (99^th^ centile).

Over time, he was noted to have global developmental delay. He sat by himself at 1 year of age. He could walk with the aid of a walker at 22 months of age. He learned to crawl at 2 years of age and walked independently at 2 years 3 months of age. He transferred objects from one hand to another at 8–10 months of age, fed himself with his hands at 15 months of age and held a spoon at 2.5 years of age. He began to coo at 18 months of age and by 3 years of age could say some phrases and could echo words, but was not speaking in sentences. A sedated auditory brainstem response (ABR) evaluation and a brain MRI performed at approximately 21 months of age were normal. He is currently enrolled in a developmental preschool program and receives speech and physical/occupational therapy.

Other medical problems encountered by subject 1 include: congenital muscular torticollis which resolved spontaneously by 2 years of age, laryngomalacia for which he underwent supraglottoplasty at 6 months of age, a right-sided cryptorchidism and a right-sided inguinal hernia which were corrected surgically at 10 months of age, gastroesophageal reflux disease that was treated medically until he was 2 years 5 months of age, and food allergies diagnosed at 1-year of age when he had anaphylaxis after eating eggs.

**Fig 3:**
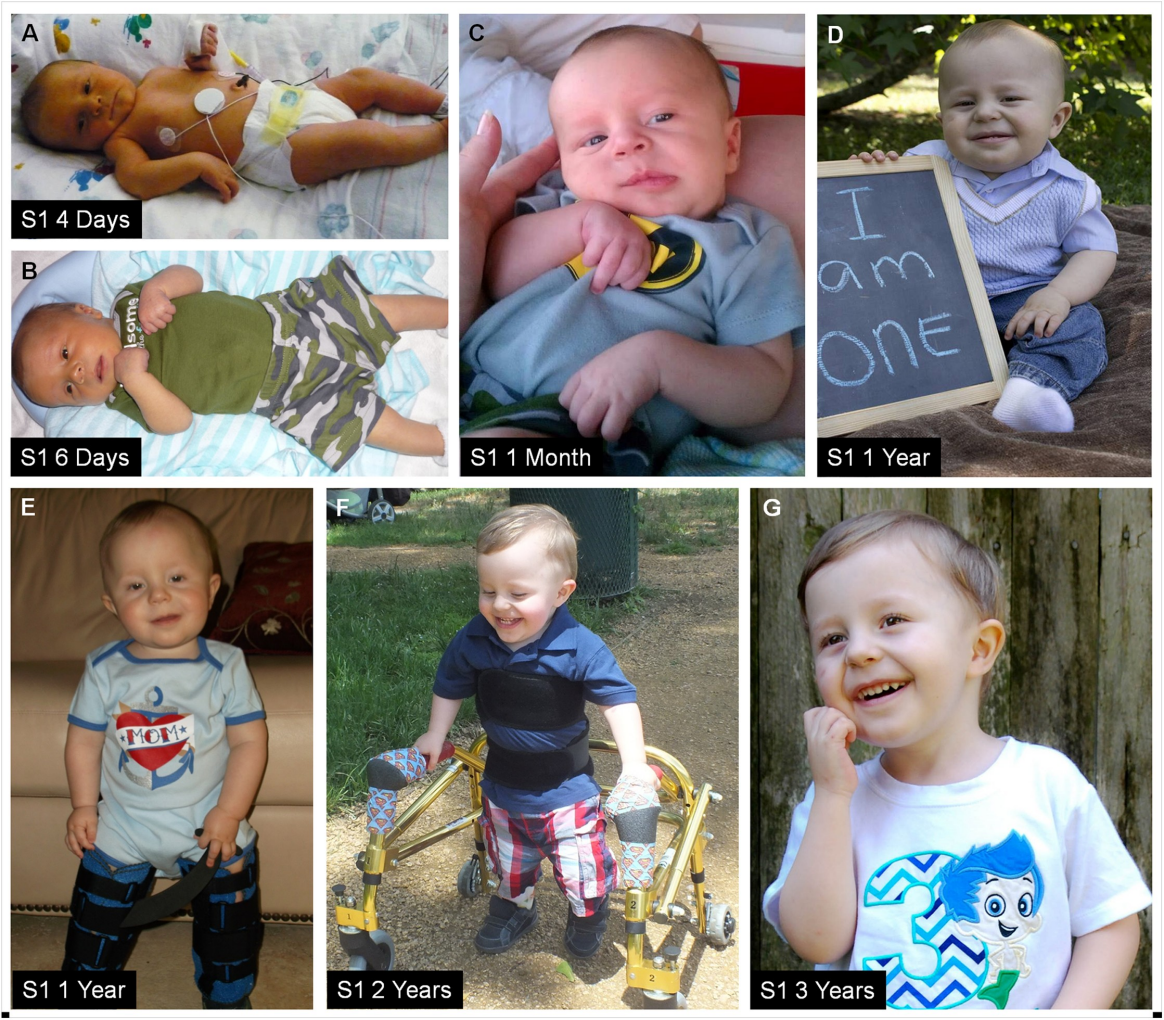
Photos of subject 1 at various ages. Subject 1 at (A) 4 days, (B) 6 days, (C) 1 month, (D-E) 1 year, (F) 2 years, and (G) 3 years of age. He does not have dysmorphic features. His congenital muscular torticollis resolved by 2 years of age. He had gross and fine motor delay attributable, at least in part, to his joint hypermobility. He could sit independently by 1 year of age (D) but required bracing to stand (E). At 22 months of age he could walk with the aid of a walker (F) and by 2 years 3 months of age he could walk independently.

At 3 years 8 months of age, his height was 95.1 cm (11^th^ centile), his weight was 13.3 kg (7^th^ centile), and his occipital frontal circumference (OFC) was 51.5 cm (85^th^ centile). On exam he was found to have relatively small testes, a hypoplastic scrotum, a sandal gap, bilateral medial malleolar displacement and joint hypermobility with a Beighton hypermobility score of 8/9 with hip hypermobility not being tested due to patient’s inability to follow commands [33]. Dysmorphic features were not noted (Fig 3).

**Fig 4:**
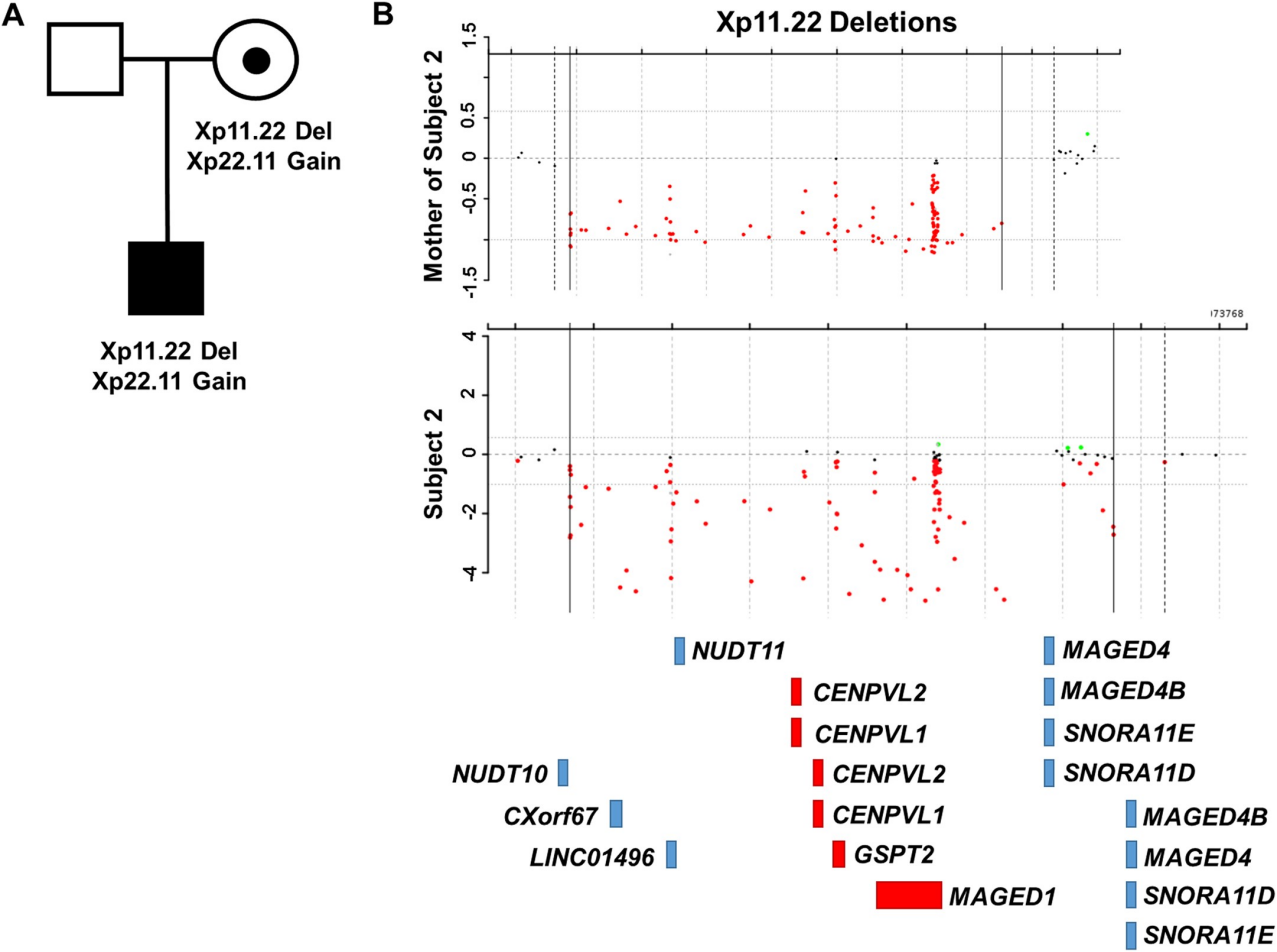
Pedigree and array-based CNV analysis for subject 2. (A) Subject 2 inherited an interstitial Xp11.22 deletion from his asymptomatic mother. (B) Data from the array-based CNV analyses performed on subject 2 and his mother. The approximate locations of the RefSeq genes in this region are shown below. Genes depicted in red are those found in the maximal overlapping region encompassed by the deletions of subjects 1–4. Genes depicted in blue were deleted in a subset of subjects 1–4.

Subject 2 is a 7-year-9-month-old male of Asian Indian descent who carries an Xp11.22 deletion (minimum deletion chrX:51,079,343–51,912,188; maximum deletion chrX:51,079,341–51,990,483; hg19) as shown in Fig 4. He also carries an Xp22.11 gain (minimum gain chrX:23,721,850–23,820,741; maximum gain chrX:23,721,848–23,850,533; hg19) that involves two genes—*ACOT9* and *SAT1* [OMIM # 300862; 313020]. Both of these changes were inherited from his asymptomatic mother.

*ACOT9* and *SAT1* have not been implicated in human disease. *ACOT9* encodes Acyl-CoA thioesterase 9, a protein that hydrolyze Coenzyme A esters [34]. Since only a part of *ACOT9* is duplicated, this could lead to a loss of ACOT9 function. We note, however, that *ACOT9* has a very low probability of loss-of-function intolerance in the ExAC database (http://exac.broadinstitute.org/; pLI = 0.0) [35]. Hence is it unlikely that haploinsufficiency of this gene would be deleterious. The entire *SAT1* gene is duplicated. This gene encodes spermidine/spermine N1-acetyltransferase, a rate-limiting enzyme in the catabolic pathway of polyamine metabolism [36]. Overexpression of SAT1 in mice leads to permanent hair loss at the age of 3 to 4 weeks and female infertility due to ovarian hypofunction and hypoplastic uteri [37]. Taken together, these findings suggest that the Xp22.11 gain carried by subject 2 is unlikely to be causative.

Subject 2 was conceived naturally by non-consanguineous parents. Pregnancy was complicated by poor maternal weight gain and concern for poor fetal growth starting at 33 weeks gestation. He was born via normal vaginal delivery at 39 weeks gestation. At birth, he weighed 2.95 kg (20^th^ centile) was 52 cm long (87^th^ centile) and had an OFC of 33.5 cm (22^nd^ centile). He was noted to have central hypotonia and was difficult to feed.

At one month of age he was diagnosed with gastroesophageal reflux disease for which he continues to be followed by a gastroenterologist. Over time, he was noted to have developmental delay. He sat at 8 months of age, pulled to a stand at 17 months of age and began walking at 24 months of age. He had foot and ankle weakness/instability requiring the use of ankle foot orthoses (AFOs). Weakness in his hands was manifest in difficulty in writing letters at school.

At 33 months of age his developmental age was estimated to be 17 months. At 3 years 4 months of age he had less than a 100-word vocabulary and was not using phrases. More recent assessments have shown a statistically significant difference between verbal skills at 76 (5^th^ centile) and nonverbal reasoning 92 (30^th^ centile) as assessed by the Differential Ability Scales-2^nd^ Edition. At 6 years of age, his adaptive behavior, social-emotional, and cognitive scores, as determined by the Developmental Profile-3, were quite low at 2%, 2%, and 0.1% respectively. He was originally enrolled in a developmental preschool but ultimately transitioned into mainstream classes. He is currently in the second grade but continues to receive occupational and speech therapy. A brain MRI obtained at three years of age was normal.

At 7 years 9 months of age his height was 119.2 cm (10^th^ centile), his weight was 18.5 kg (<1^st^ centile, Z score = -2.40) and his OFC was 54.5 cm (96^th^ centile). He had diffuse, profound hypotonia, was still using AFOs, had pes planus, and had bilateral medial malleolar displacement. He was noted to have joint hypermobility and facial weakness with a tented mouth but did not have dysmorphic features. He had normal deep tendon reflexes. He made excellent eye contact but struggled to execute simple commands and had very limited expressive language.

Subjects 3 and 4 are full brothers who both carry ~1.5 Mb Xp11.22 deletions (chrX:51,357,052–52,838,176, hg19) that they inherited from their asymptomatic mother. Subject 3 is described in the DECIPHER database as patient 276715. Subject 3 and 4 were conceived naturally by non-consanguineous parents. They have an older, paternal half-brother who has no medical problems. A deceased maternal great uncle was reported to be “handicapped” but further details regarding his condition are not available.

Subject 3 is a 6-year-old male who was born at 42 weeks gestation. His birth weight was 3.3 kg (50^th^ centile). He started walking at 27 months of age and talked at 36 months of age. He has mild hypermetropia and intermittent exotropia, hypotonia, joint hypermobility and arthralgias. He does not have dysmorphic features. At 6 years of age, his height was at the 0.4^th^ centile, his weight at the 2^nd^ centile, and his OFC at the 50^th^ centile.

Subject 4 is a 4-year-old male who was born at 36 3/7 weeks of gestation by emergency cesarean secretion. His birth weight was 2.7 kg (39^th^ centile). He started talking at 24 months of age and walking at 30 months of age. He had exotropia and was treated for amblyopia. He has epicanthal folds but does not have additional dysmorphic features. He has a single capillary nevus. He has pes planus for which he uses orthotics. At 4 years of age his height was at the 2^nd^ centile, his weight at the 25^th^ centile and his OFC at the 75^th^ centile.

## Discussion

### The phenotypes of subjects 1–4 define a distinct Xp11.22 deletion syndrome

Subjects 1–4 subject share a core set of phenotypes that include intellectual disability, developmental delay, hypotonia, joint hypermobility and abnormal growth patterns that include short stature, failure to thrive and relative macrocephaly. Each was found to carry an Xp11.22 deletion that they inherited from their asymptomatic mothers. The maximum region of overlap of subjects 1–4’s deletions spans ~430 kb (chrX:51,357,052–51,786,912; hg19). This critical region contains two pseudogenes, *CENPVL1* and *CENPVL2*, whose functions are not known, and two protein coding genes, *GSPT2* and *MAGED1* (Fig 1). Deletions involving *CENPVL1*, *CENPVL2*, or the coding regions of *GSPT2* and *MAGED1* were not observed among control individuals cataloged in the Database of Genomic variants (http://dgv.tcag.ca/dgv/app/home).

The Xp11.22 deletions documented in subjects 1–4 did not include any of the Xp11.22 genes whose deficiency has been previously implicated in the development of intellectual disability/developmental delay or whose deficiency has been hypothesized to contribute to neurocognitive disorders based on human studies [1–14,18,19,24–27]. Specifically, *SHROOM4* is located telemetric to these deletions and *TSPYL2*, *KDM5C*, *IQSEC2*, *HUWE1*, *PHF8* and *FAM120C* are located progressively centromeric to these deletions (Table 2).

**Table 2:** Coordinates of Xp11.22 deletions and Xp11.22 genes previously implicated or proposed to be involved in the development of intellectual disability/developmental delay/autism.

Xp11.22 Deletion or Gene	Start (Maximum) hg19	End (Maximum) hg19
*SHROOM4*	50,334,643	50,557,044
Deletion in subject 1	50,789,912	51,786,912
Deletion in subject 2	51,079,341	51,990,483
Deletion in subjects 3 and 4	51,357,052	52,838,176
Critical region based on subjects 1–4	51,357,052	51,786,912
*TSPYL2*	53,111,549	53,117,723
*KDM5C*	53,220,503	53,254,604
*IQSEC2*	53,262,058	53,310,796
*HUWE1*	53,559,057	53,713,674
*PHF8*	53,963,113	54,071,569
*FAM120C*	54,094,757	54,209,714

It is currently unclear whether deletion of the ~430 kb critical region on Xp11.22 defined by subject 1–4 constitutes a contiguous gene deletion syndrome, in which the associated constellation of phenotypes is due to the loss of two or more genes, or if the loss of a single gene within the critical region is sufficient to cause all of these phenotypes.

### Loss of *CENPVL1* and *CENPVL2* as a potential contributor to the phenotypes seen in subjects 1–4

*CENPVL1*, *CENPVL2* are pseudogenes of the centromere protein V gene (*CENPV*) located on chromosome 17p11.2 [OMIM #608139]. CENPV is a kinetochore protein whose deficiency in cells leads to defects in chromosome alignment in metaphase, lagging chromosomes in anaphase, failure of cytokinesis, and apoptotic cell death [38]. CENPV also plays a role in directional cell motility and acts as a scaffolding molecule that links microtubules and Src family kinases [39].

Although it is unclear whether *CENPVL1*, *CENPVL2* are required for normal development, a growing body of evidence suggests that pseudogenes may perform a variety of biological functions including the regulation of their parent genes [40–44]. Hence, it is possible that loss of *CENPVL1* and *CENPVL2* may contribute to the phenotypes of subjects 1–4.

### Loss of *GSPT2* as a potential contributor to intellectual disability and developmental delay

GSPT2 is widely expressed with relative abundance in brain [45]. Whibley et al. suggested that *GSPT2* was a candidate gene for X-linked intellectual disability after identifying a maternally inherited tandem duplication of *GSPT2* in two brothers with intellectual disability [46]. The effects of GSPT2 deficiency on CNS function have not been demonstrated in humans or studied in mice. However, *GSPT2* has a high probability of loss-of-function intolerance (pLI = 0.9) with no loss-of-function variants being seen among controls catalogued in the ExAC database even though 7.3 loss-of-function variants were expected [35].

GSPT2 encodes a GTPase that belongs to the GTP-binding elongation factor family. GSPT2 complexes with eukaryotic peptide chain release factor 1 to mediate translation termination and prevent the translation of an extended protein product (readthrough) [47,48]. This suggests that loss of GSPT2 may lead to the production of abnormally elongated proteins which might, in turn, be detrimental to cells. Since GSPT2 is more abundantly expressed in the brain, its loss may have greater effects on these cells.

Xiao et al. have shown that GSPT2 also interacts with BIRC5 (also known as survivin), an antiapoptotic protein that plays an essential role in early brain development [49,50]. They suggest that GSPT2 and BIRC5 may function cooperatively in nuclear processes. However, ablation of *Birc5* in the neuronal precursor cells of mouse embryos starting at E10.5 leads to reduced brain size associated with severe, multifocal apoptosis in the cerebrum, cerebellum, and brainstem—a phenotype not seen in our subjects who have relative macrocephaly and, in the case of subjects 1 and 2, normal brain MRIs [49,50].

### Loss of *MAGED1* as a potential contributor to intellectual disability and developmental delay

A search of the ExAC database suggests that there is a paucity of loss-of-function variants in *MAGED1* with only one loss-of-function being observed and 16.4 being predicted. This gives MAGED1 a high probability of loss-of-function intolerance (pLI = 0.98) [35]. Although it is unclear how loss of MAGED1 function affects humans, experiments performed in mice suggest that loss of MAGED1 may result in neurocognitive and/or neurobehavioral abnormalities.

Bertrand et al. demonstrated that MAGED1 is highly expressed throughout the brain particularly in the striatum, the hippocampus, the incipient dentate gyrus, the thalamus, the hypothalamus, the olfactory bulb and in the neuroepithelium and subventricular zone of the cerebral cortex [51]. Subsequently, Yang et al. used behavioral tests and electrophysiological recording to show that 5–6-month-old *Maged1*-null mice displayed reduced basal synaptic transmission, pronounced hippocampal dysfunction, impaired spatial learning, and a deficit in long-term potentiation induction [52]. They also showed that loss of MAGED1 resulted in a reduced dendritic spine density and a reduction in the number of synapses in the hippocampi of *Maged1*-null mice. They concluded that MAGED1 plays a role in synaptic transmission and hippocampus-dependent learning and memory function.

Dombret et al. demonstrated that a lack of MAGED1 function in male mice results in deficits in social interactions [53]. Specifically, these mice also had reduced ultrasonic vocalization responses to females in estrus, reduced direct social interactions with normal social preference, reduced exploratory behavior, impaired social memory or no preference for social novelty, increased self-grooming and increased anxiety. Dombret et al. went on to shown that although the hypothalami of *Maged1*-null males were normally formed, their hypothalamic mature oxytocin levels were lower than in wild-type males, possibly due to defective oxytocin post-translational processing. Interestingly, an acute subcutaneous oxytocin injection rescued the impaired social recognition of MAGED1-deficient male mice. They concluded that a lack of MAGED1 function could play a role in autism.

Mouri et al. demonstrated depression-like behavior in *Maged1*-null mice such as decreased social exploratory behavior, decreased social interaction, increased immobility time during forced swimming and tail suspension, and a decrease in sucrose preference [54]. They found that acute and chronic administration of sertraline and imipramine reversed all or part of the depression-like behavior in these mice. They concluded that a lack of MAGED1 function could play a role in the development of depressive behaviors.

The molecular mechanisms by which MAGED1 affects brain development and function have not been fully elucidated. However, Sullivan et al. demonstrated that MAGED1 binds and positively regulates the transcriptional activity of a subset of transcription factors in the basic helix-loop-helix (bHLH) PER-ARNT-SIM (PAS) family [55]. This subset includes SIM1, NPAS4 and ARNT2 whose deficiencies have been shown to affect brain development/function [55–58].

### Phenotypes seen in MAGED1-deficient mice which are not currently evident in subjects 1–4

MAGED1-deficient mice develop progressive obesity associated with hyperphagia and reduced motor activity [53]. Obesity is not seen subjects 1–4. In contrast, short stature and failure to thrive are common. Maged1-deficient mice also have a significant osteoporotic phenotype with a marked decrease in bone density and deterioration of trabecular architecture [53]. The bone densities of subjects 1–4 have not been measured, but none have a history of recurrent or pathogenic fractures. It is possible that these phenotypes will become apparent in subjects 1–4 over time. Alternatively, these phenotypes may be absent due to inherent differences between mice and humans or due to the compensatory effects of the reduced function of other genes in the Xp11.22 critical region.

## Conclusions

These results suggest that deletions of an ~430 kb region on chromosome Xp11.22 that encompass *CENPVL1*, *CENPVL2*, *GSPT2* and *MAGED1* cause a distinct X-linked syndrome characterized by intellectual disability, developmental delay, hypotonia, joint hypermobility and relative macrocephaly. Loss of GSPT2 and/or MAGED1 function may contribute to the intellectual disability and developmental delay seen in males with these deletions.

## Supporting information

S1 Table PCR confirmation and fine mapping of the Xp11.22 deletion of subject 1. (PDF)
