# Supplementary material for: Xp11.22 deletions encompassing CENPVL1, CENPVL2, MAGED1 and GSPT2 as a cause of syndromic X-linked intellectual disability
Source: PLoS One. 2017 Apr 17;12(4):e0175962. doi: 10.1371/journal.pone.0175962 (PMC5393878; doi:10.1371/journal.pone.0175962)
Supplement: S1 Table — (PDF) [file pone.0175962.s001.pdf]

Table S1: PCR confirmation and fine mapping of the Xp11.22 deletion of subject 1

Interval Amplified (hg19)	Primer Pair Name	Primer Pair (Forward/Reverse)	Amplicon Size (Base Pairs)	Amplified from subject 1?
chrX:50,729,647-50,729,848	Sub 1 Xp11.22 5' pos control	5'-AAGAACACTGCTGGAACTTTGG-3'<br>5'-TGCAGCCTTTGTGTTAATGCT-3'	201	Yes
chrX:50,743,151-50,743,384	Sub1 Xp11.22 5' A	5'-ACAATGTAGGCTTTAGCACAACA-3'<br>5'-GGTGATGCATTGCTAGCATGT-3'	233	Yes
chrX:50,767,670-50,767,871	Sub 1 Xp11.22 5' B	5'-GGAACTTTCCTGCTGAACTAGAG-3'<br>5'-TTATGGATATTGAAATGCAGTGAGGA-3'	201	Yes
chrX:50,789,502-50,789,911	Sub 1 xp11.22 5' C	5'-TCAGCTGAGACCCTATGCCT-3'<br>5'-TGGGACAACAATTGCTATTTGTAGA-3'	410	Yes
chrX:50,846,687-50,847,688	Sub 1 Xp11.22 5' min neg control 5'	5'-CATCATTACCCCTGCTGTGC-3'<br>5'-GATGCACTCATCCCCACTCA-3'	101	No
chrX:51,731,836-51,731,951	Sub 1 Xp11.22 3’ min neg control 3’	5'-ACTGCTCAGCGTTAGGGAAG-3'<br>5'-TGCCCACCTTTGACACCAAT-3'	115	No
chrX:51,773,705-51,773,907	Sub 1 Xp11.22 3’ A	5'-GCCCCAAACTCTCCAGTTCT-3'<br>5'-CCTCTCATCAACAAACGTTCACC-3'	202	No
chrX:51,786,912-51,787,145	Sub 1 Xp11.22 3' B	5'-CCCCAGCAAGCCTTTCCTT-3'<br>5'-AGGCGCACAAAATATTTCCGA-3'	233	Yes
chrX:51,805,406-51,805,640	Sub 1 Xp11.22 3' pos control	5'-CATCTAGGACGCTGGTGCTG-3'<br>5'-ATATCGGGGATGAAGCGCTG-3'	234	Yes
